# Multipurpose vaginal rings: preferences from a national discrete choice experiment survey among US women

**DOI:** 10.3389/frph.2026.1722593

**Published:** 2026-02-19

**Authors:** Ann Gottert, Sanyukta Mathur, Barbara A. Friedland, Timothy Abuya, Irene V. Bruce, Brady Burnett-Zieman, Marlena G. Plagianos, Shakti Shetty, Michelle Nguyen, Jessica M. Sales, Matthew Quaife, Lisa B. Haddad

**Affiliations:** 1 Population Council, Washington, DC, United States; 2 Population Council Center for Biomedical Research, New York, NY, United States; 3 Independent Consultant, Nairobi, Kenya; 4 Department of Behavioral, Social, and Health Education Sciences, Emory University, Atlanta, GA, United States; 5 Evidera, London, United Kingdom; 6 Department of Infectious Disease Epidemiology, London School of Hygiene and Tropical Medicine, London, United Kingdom

**Keywords:** contraception, discrete choice experiment, HIV, intravaginal rings, multipurpose prevention technologies, nonhormonal, sexually transmitted infections

## Abstract

**Background:**

We assessed US women's preferences to inform development of a novel nonhormonal multipurpose prevention technology (MPT)—a vaginal ring to prevent pregnancy, HIV, sexually transmitted infections (STIs), and bacterial vaginosis (BV).

**Methods:**

In cross-sectional online surveys with US women ages 18–49 currently/interested in using contraception, we conducted a discrete choice experiment (DCE) comprising 7 MPT ring attributes. Mixed multinomial logit models examined relative attribute importance and sub-population preferences.

**Results:**

Of 2,105 survey completers (mean age 31) from all 50 states (Dec 2023 to Jan 2024), 53% were married/cohabiting, 57% had ≥1 child, 43% ever had an unintended pregnancy, and 9% had an STI in the past year. Participants valued effectiveness for contraception about twice as much as for HIV prevention and about 3 times that of STI prevention. Younger women (18–29 vs. 30–49) desired higher pregnancy and HIV prevention effectiveness. Women who were worried about HIV valued effectiveness for HIV and pregnancy similarly. While most women valued BV prevention and no menstrual side effects, a nonhormonal formulation mattered only to women averse to hormonal contraception (61%) and on-demand use (vs. continuous-use only) was not preferred. Women were willing to trade off some pregnancy prevention effectiveness for other desired attributes. Overall, 73% reported being likely/very likely to use a nonhormonal MPT ring at moderate protection levels (80% pregnancy, 50% HIV/STIs).

**Conclusions:**

Interest in an MPT ring was strong, even with conservative effectiveness estimates. Preferences and desired levels of prevention effectiveness and nonhormonal options were shaped by contraceptive history and personal context.

## Introduction

Multipurpose prevention technologies (MPTs) could help meet women's varied sexual and reproductive health needs, including protection against pregnancy, HIV, and/or other sexually transmitted infections (STIs). Despite the availability of highly effective single-purpose products, unmet need for pregnancy and HIV/STI prevention remains high. Globally, over 40% of all pregnancies are unintended (1), as are nearly half in the US (2). Most (95%) unintended pregnancies occur among women not using their contraceptive method correctly, consistently, or at all (3), and nearly 4 in 10 of women discontinue a method within 12 months of initiation despite wanting to prevent or delay pregnancy (4). An estimated 1.3 million people acquire HIV each year, well beyond global prevention targets, with suboptimal uptake and continuation of effective biomedical prevention options to date, such as oral pre-exposure prophylaxis (PrEP), including among women (5). Other STIs like syphilis, gonorrhea, and chlamydia pose broad-reaching risks particularly for women and their children, including the increased risk of HIV acquisition and transmission, chronic pelvic pain, infertility, and preterm delivery, and are surging in the US and globally, with over one million new infections each day (6–8).

Women worldwide, including in the US, consistently express interest in MPTs (9–12). A recent global survey found that 83% of women preferred HIV/STI prevention products with contraception vs. HIV/STI prevention alone (12). By combining several indications in a single product, MPTs can increase convenience and accessibility, reduce stigma often associated with HIV/STIs, improve adherence, and enhance health outcomes and quality of life (13, 14).

Vaginal rings are the most common MPT delivery form in development (8 of 21 products) (15). Vaginal rings are long-acting, with local activity and minimal systemic release of active pharmaceutical ingredients (APIs), user-controlled (women insert and remove them themselves) and require limited clinical training or special equipment. Several single-indication contraceptive rings are available on the US market, including NuvaRing® (including its generic versions) and Annovera® (16). An HIV prevention ring, DapiRing, is also on the market in 13 African countries (17). Studies worldwide show that most women who use vaginal rings find them highly acceptable (as do their male partners), with high continuation rates (18–25). Current efforts to develop MPT vaginal rings are all in early/preclinical stages.

Early research with end-users is critical to inform MPT product design and target product profiles (14, 26), and to help ensure that the development and introduction of MPTs can be optimized to meet women's sexual and reproductive health (SRH) needs. Understanding how end-users prioritize and make tradeoffs between varied potential MPT attributes is critical to ensuring future acceptability and choice. Almost all existing research with women about potential interest in/acceptability of MPT rings has focused specifically on continuous-use rings that combine hormonal contraception with ARV-based HIV prevention (6, 22, 27). While most of the eight MPT rings in development are designed to prevent pregnancy and HIV only (15), some also prevent other STIs and/or bacterial vaginosis (BV). These candidate vary substantially in hormone content, active ingredients (e.g., antiretrovirals, copper, etc.), effectiveness across indications, potential side effects and menstrual changes, and use regimens (e.g., continuous vs. on-demand). This diversity highlights the need to better understand how end-users prioritize and make tradeoffs among multiple MPT ring attributes. Moreover, established frameworks in reproductive decision-making, discrete choice theory, and person-centered frameworks (28–32) suggest that preferences are likely to differ among different subgroups of women, such as those with higher vulnerability to unintended pregnancy or HIV/STIs, or those preferring nonhormonal options. Such heterogeneity remains underexplored in vaginal ring acceptability/preference research to-date (19, 20).

In this study, we explored US women's preferences regarding MPT rings, to contribute to the field's understanding and to inform the design of a novel nonhormonal MPT ring our team is developing to prevent pregnancy, HIV, several other STIs, and BV (5P50HD106793; PI: Haddad) (33). We set out to determine (1) women's preferences regarding key attributes of an MPT ring, via a discrete choice experiment (DCE), (2) acceptability of a nonhormonal MPT ring product; and (3) heterogeneity in product preferences and acceptability among women with differing characteristics.

## Methodology

We conducted an online survey with US women aged 18–49 years who were currently using or interested in using contraception. Recruitment was managed by CloudResearch®, an online research platform. Participants were recruited via Prime Panels (34, 35), an aggregation of opt-in market research panels that are commonly used for online research where people perform tasks for a nominal fee, including completing surveys. In Prime Panels, participants first complete a brief screener to identify and block individuals who provide poor quality responses (36–38). Such online research panels have been used extensively in social and behavioral science research, including studies on MPT interest (35, 39, 40).

We targeted a sample size of 2,000 to ensure adequate statistical power for the DCE (41), and to allow the examination of preference heterogeneity by several different characteristics (e.g., age group, parity, HIV/STI risk perception). Eligible participants were female, aged 18–49 years, living in the US, reporting current contraceptive use or interest in contraceptive use in the next five years; sexual intercourse (penile-vaginal) with a male partner within the last year; and no tubal ligation or hysterectomy. We set quotas within Prime Panels (based on the existing information in that platform) to ensure 50% of the participants were aged 18–29 to ensure we captured sufficient numbers of both younger and older women, and that at least 30% were Black/African American as evidence suggests that preferences for different SRH product attributes (e.g., STI protection, on-demand use, contraceptive efficacy) vary across ethno-racial groups in the US (30), and African-American women face a disproportionate burden of unintended pregnancies and HIV/STIs (2).

The survey was programmed and hosted on REDCap (42, 43), and available in English or Spanish. Prime Panels sent eligible respondents a link to the survey, titled “Contraceptive Preferences Survey” estimated to take 20–30 min. Respondents were asked to read through an informed consent form and indicate their consent before proceeding with the survey. Participants who completed the survey were compensated at a rate pre-set by Prime Panels (≤$10).

### Conceptual framework

**Figure 1:**
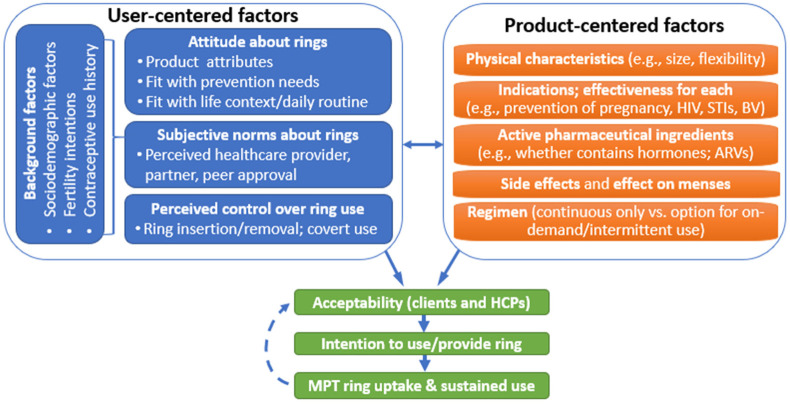
Conceptual framework of user- and product-centered factors influencing vaginal ring acceptability.

This study was guided by a conceptual framework (Figure 1) in which product- and user-centered factors interact to influence user acceptability, intention to use, and ultimately, product use. Drawing on prior frameworks (44, 45), product-centered factors include physical characteristics, indications, active pharmaceutical ingredients (APIs, e.g., contains hormones), side effects, effects on menses, and regimen. Specific user-centered factors were informed by the Theory of Planned Behavior (46) in which three determinants influence behavioral intentions: (1) attitude (e.g., about contraception, HIV, and STI prevention, and the product attributes), (2) subjective norms (e.g., perceived healthcare provider, partner, or peer approval of product use), and (3) perceived behavioral control (e.g., over ring insertion/removal, or adhering to regimen). Participant background factors (e.g., sociodemographic characteristics, reproductive and contraceptive history) are hypothesized to influence these determinants. Building on this conceptual framework, and informed by a review of the literature as well as formative in-depth interviews with women (described below), we hypothesized that participants would express clear preferences regarding MPT ring product attributes. Specifically, we anticipated positive preferences for higher effectiveness for each indication (with an overall pattern of higher preference for pregnancy prevention over other indications), no impact on menses, and nonhormonal and on-demand options. We further hypothesized that these preferences, and overall interest in the product, would vary to some extent according to user-centered factors most directly related to these attributes, such as perceived prevention needs for each indication, attitudes toward hormones, prior experiences with contraceptive side effects or menstrual changes, and familiarity or comfort with vaginal rings.

### DCE design

The main theoretical estimands (47) of interest in this study were women's relative preferences for MPT ring product attributes, and heterogeneity in those preferences. To approximate these estimands empirically, we conducted a DCE, an experimental survey method that quantifies how individuals value different product attributes by observing the trade-offs they make between alternative options, that also allows for the examination of preference heterogeneity (48). In DCEs, respondents are often asked to complete a series of “choice sets” in which they choose between Option A or Option B, each with varying levels of the same attributes (41, 48, 49). Following best practice recommendations (41, 50), we began by identifying potentially salient DCE attributes such as effectiveness for pregnancy prevention or potential effect on menses, as well as levels for each of these attributes, such as specific levels of effectiveness or the nature of effect on menstrual cycle. We first reviewed the potential characteristics of the nonhormonal MPT ring under development, and conducted a rapid literature review of end-users' opinions about MPTs and vaginal ring products. We also conducted in-depth interviews which included a card-sort exercise with 25 women recruited via ResearchMatch. Additional qualitative findings are described elsewhere (51).

Next, based on the final DCE attributes and levels, we selected 12 choice sets from all possible choice sets, via an experimental design using NGENE software (52) that employed a fractional factorial approach (41, 53). We limited to 12 to minimize cognitive burden that can lead to respondent fatigue (48). A graphic designer created visuals to accompany or replace wording in the choice sets to improve comprehension. We informally pre-tested the choice set layout, wording and visuals with 10 women, using cognitive interviewing techniques, and tested the functionality and readability of choice sets on various potential devices (such as computer, tablet, smartphone). Finally, we pilot-tested the survey with 49 respondents via Prime Panels. We generated final choice sets in NGENE using a d-efficient design algorithm [which yields the most statistically efficient parameter estimates given the sample size (48, 53)], based on priors from the pilot data. The DCE design is included in Supplementary File 1. We programmed the survey to randomize both the order of attributes within choice sets and the order of choice sets across respondents, to minimize positional bias.

**Figure 2:**
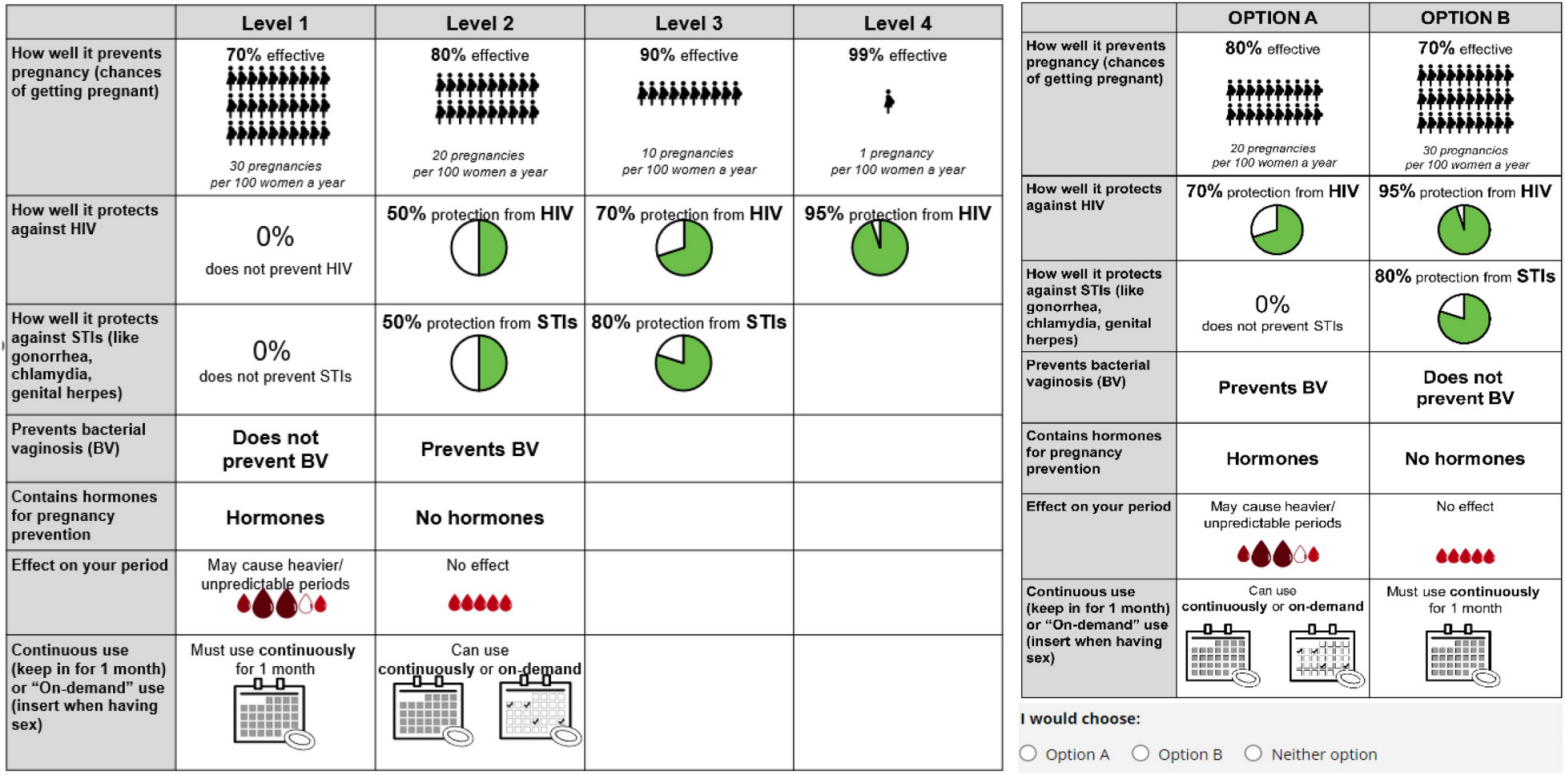
DCE attributes/levels from which choice sets were selected (left), and an illustrative choice set (1 of 12, right).

Figure 2 presents the final attributes and levels for each that were the basis for the 12 final choice sets, as well as an illustrative choice set. Before completing the DCE choice sets, women were asked to read introductory text about the MPT ring under development, what vaginal rings are, their use, and currently available vaginal rings in the US. Respondents were then asked to review the seven ring attributes included in the choice sets. The description of the “Contains hormones” attribute noted that “For a ring that does NOT contain hormones, the active ingredients could be copper or zinc, which have been used in contraceptives before. These ingredients will prevent sperm from fertilizing an egg.” Full introductory text and selected visuals are in Supplementary File 2. Participants then proceeded with choosing “Option A”, “Option B”, or “Neither option” for each of the 12 choice sets. If they chose “Neither option” for a given choice set, they were asked a forced choice question: “For the scenario above, if you HAD to choose between Option A or Option B, which would you choose?”

### Additional measures

After the DCE choice sets, we asked participants directly about how likely they would be to use a vaginal ring with a specific set of attributes (based on anticipated attributes of the nonhormonal MPT ring in development, with moderate levels of effectiveness), as well as several follow-up questions about the nature of and reasons for anticipated use. We also asked a range of questions related to socio-demographic characteristics, fertility intentions, reproductive and contraceptive history, HIV/STI history and risk perception, perceived behavioral control over potential ring use, and subjective norms around potential ring use.

### Data analysis

We analyzed all data using Stata v18. We generated descriptive statistics (means/frequencies and measures of dispersion) for socio-demographic characteristics and other key study variables. We screened for low data quality by identifying instances in which a participant completed the survey in under 3 min, and/or provided the same response for all 12 DCE choice sets question (i.e., all option A, all option B, or all Neither option) and for at least two of three other pre-specified blocks of questions (contraceptive method use history, HIV prevention method use history, and importance of different aspects of contraceptive methods).

For the DCE analysis, participants' choices were the dependent variable and the attribute levels within the choice sets were the independent variables. The DCE model estimates utilities under the assumption of stable choice consistency and independence of irrelevant alternatives (48). We first used a conditional logit model and inspected several model diagnostic indicators including opt-out bias. While the conditional logit DCE model results showed that alternative specific constant (ASC) for the opt-out (selection of “neither option”) was positive and significant (*p* < 0.05), suggesting that participants preferred to opt out, in the subsequent mixed multinomial logit (MMNL) model (48) the ASC was negative and statistically significant (*p* < 0.001), suggesting that participants preferred to opt in. We therefore decided not to use the forced choice responses.

For subsequent DCE models, we used MMNL models to account for heterogeneity of preferences across respondents and for within-respondent correlation (54). We used 1,000 Halton draws [a method of generating quasi-random numbers to be used in simulation-based estimation of the model, that can yield more accurate approximations with fewer draws, speeding up estimation (53)], with a normal distribution specified for all attributes. We generated mean preference weight estimates, with 95% confidence intervals (CIs), for each attribute in the MMNL model. For all DCE analyses, we modeled pregnancy, HIV, and STI prevention effectiveness as continuous variables (ranging from 0% to 100%), since the underlying attributes are naturally ordered and anticipated to be roughly continuous in the real world, and continuous coding enables calculation of marginal utilities/willingness to pay (i.e., the % effectiveness respondents would give up for other desired attributes). For continuously coded attributes, mean preference weights indicate preference for a 1% increase. All other attributes were binary variables and were effects coded. In effects coding, zero represents the mean effect across all levels of the variable, rather than the effect for the omitted level (as is the case for dummy coding). Therefore, mean preference weights reflect preference for a given attribute level relative to the mean attribute effect, with positive coefficients reflecting greater, and negative coefficients lower, preference than average effects.

We calculated the relative importance of each attribute in relation to the other attributes, using the means from the MMNL model. We multiplied the absolute value of the mean of each attribute's parameters by the difference between the attribute levels' highest and lowest values to give the maximum effect. We calculated relative importance values by considering the proportion of the maximum effect in the context of the total for each attribute.

We also estimated the tradeoffs (alternately referred to as willingness-to-pay, or marginal rates of substitution) that women were willing to accept in pregnancy, HIV, and STI prevention effectiveness (i.e., the continuously coded attributes) for each other attribute using the delta method to estimate the standard errors and confidence intervals for willingness-to-pay estimates derived from mixed logit models (55). For tradeoffs with other continuously coded effectiveness attributes, to improve interpretability we multiplied the value by 10 to represent the % reduction in effectiveness for one indication (e.g., pregnancy prevention) women would forego for a 10% increase in effectiveness for another indication (e.g., HIV prevention).

We assessed heterogeneity in DCE preferences for a series of respondent characteristics by which preferences were hypothesized to vary, each of which was constructed as a binary variable. First, we added each interaction term between a given attribute and a respondent characteristic (such as HIV prevention effectiveness and age group) into the conditional logit model one-by-one. Each statistically significant interaction term from that model was then entered, one-by-one, into the MMNL model, with coefficients for each interaction term representing the difference in mean preference weight for that attribute between women with vs. without that given characteristic.

We also conducted exploratory sub-group analyses, based on statistically significant interaction terms. These analyses entailed generating mean preference weights for certain attributes, and willingness to accept reductions in pregnancy/HIV/STI prevention effectiveness for these attributes. An example is, among women for whom nonhormonal contraception is very important, generating the mean preference weight for a nonhormonal ring option (vs. hormonal), and what % reduction in pregnancy prevention effectiveness those women would be willing to accept for the ring to be nonhormonal.

Finally, to examine heterogeneity in reported likelihood of using a nonhormonal MPT ring product (assessed via the direct elicitation question) we conducted bivariate and multivariate ordinal logistic regression analyses, with the outcome coded as (0) Very unlikely/unlikely/don't know; (1) Likely; (2) Very likely. The final multivariate model was achieved via backward elimination, removing non-significant variables one-by-one, in order of highest magnitude p-value, until all variables in the model were statistically significant (at *p* < 0.05). We believe the backward elimination approach is justified since the purpose of the model is exploratory (not causal), the set of independent variables is theoretically justified, and sample size is large relative to the number of independent variables (56). Supplementary File 3 includes a correlation matrix for variables representing characteristics of women, that were included in the MMNL interaction models above and/or the multivariate ordinal logistic regression analyses described in this paragraph, which showed low correlations, most <0.2, with the only moderate correlation (0.61) being between HIV worry and STI worry. Moreover, variance inflation factors (VIFs) for the latter analysis were all <2, indicating no concerns about multicollinearity (56).

## Results

Between December 2023 and January 2024, 8,787 women entered the eligibility/screening form and 7,533 completed it. Of those, 2,606 (35%) were eligible, 2,555 (98%) gave informed consent, and 2,111 (83%) completed the survey. Six of these respondents were removed based on the pre-specified quality screening criteria (all related to response patterns), resulting in 2,105 included in the analysis. The most common reason for ineligibility was not using and not interested in using contraception. Surveys took an average of 21 min to complete.

The mean age of participants was 31.3 years (range 18–49) (Table 1). About 62% of participants identified as White, nearly one-third (32%) as Black/African American and about 18% identified as Hispanic. Women came from all 50 US states and a mixture of urban (32%), suburban (43%) and rural (25%) settings. The largest proportion (46%) lived in the South, which is likely due to the quota over-representing African Americans, 57% of whom live in the South per the 2020 census (57). Just over half (53%) were married/cohabiting; 57% had at least one child and almost half (49%) wanted (an)other child (with varied desired timing). A minority did not want a(nother) child (28%) or were unsure (23%); 43% ever had an unintended pregnancy.

**Table 1:** Respondent characteristics, (*n* = 2,105 women).

Characteristic	*n* (%)
Age—mean (standard deviation, range)	31.3 years (8.82, 18–49)
Race (respondents could select multiple)	
** **White	1,313 (62.4)
** **Black/African American	673 (32.0)
** **American Indian or Alaska Native	62 (3.0)
** **Asian	86 (4.1)
** **Pacific Islander	13 (0.6)
** **Another race	76 (3.6)
Ethnicity	
** **Hispanic	366 (17.5)
** **Non-Hispanic	1,728 (82.5)
US region	
** **Northeast	342 (16.3)
** **Midwest	437 (20.8)
** **South	968 (46.0)
** **West	358 (17.0)
Rurality	
** **Urban	680 (32.3)
** **Suburban	905 (43.0)
** **Rural	520 (24.7)
Educational attainment	
** **Some high school or less	92 (4.4)
** **High school diploma or GED	1,053 (50.0)
** **Associates/Bachelors degree	787 (37.4)
** **Masters, Doctoral, or other Professional degree	173 (8.2)
Total combined family income (annual)	
** **Less than $50,000	1,010 (49.6)
** **$50,000 to $99,999	725 (35.6)
** **$100,000 to $199,999	251 (12.3)
** **Over $200,000	49 (2.4)
Relationship status	
** **Married and living together	544 (25.8)
** **Married but not living together	45 (2.14)
** **Unmarried, in a relationship and living together	534 (25.4)
** **Unmarried, in a relationship but not living together	449 (21.3)
** **Not currently in a relationship	533 (25.3)
Number of biological children	
** **None	912 (43.3)
** **1 child	464 (22.0)
** **2 children	418 (19.9)
** **3 or more children	311 (14.8)
Want another child	
** **Yes	1,039 (49.4)
** **No	583 (27.7)
** **Unsure	483 (23.0)
Timing of desired (next) child [among those who want a(nother) child/unsure]	
** **Within the next year	217 (14.3)
** **1–2 years from now	455 (29.9)
** **3–4 years from now	338 (22.2)
** **>5 years from now	227 (14.9)
** **Not sure	285 (18.7)
Ever had an unintended pregnancy	
** **No	1,109 (52.7)
** **Yes	898 (42.7)
** **Not sure	63 (3.0)
** **Prefer not to respond	35 (1.7)

The most common currently used contraceptive methods (Supplementary Table 1) were male condoms (43%), birth control pills (32%), withdrawal (27%), fertile days (13%), and emergency contraception (10%). The most common methods women reported using to prevent HIV were male condoms (48%) and pre-exposure prophylaxis (PrEP) (5%).

As Table 2 shows, 47% of women reported ever discontinuing a contraceptive method due to unwanted side effects, and 25% due to unwanted changes in menses. Nearly 80% had a history of menstrual problems, including very heavy bleeding, irregular menses, and/or spotting between menses. Nearly half (49%) reported ever having BV. When asked how important it was to them that their contraception be nonhormonal, 35% said very important, 25% said important, and 26% said somewhat important. In terms of experience with vaginal products, 17% had ever used a vaginal ring, 4% were currently using this method, and one-third (33%) knew others who had. Nearly all (94%) had used other types of vaginal products (e.g., tampons; sexual lubricants).

**Table 2:** Sexual and reproductive health history, practices, and preferences (*n* = 2,105 women).

Variable	*n* (%)
Contraception, menses, and vaginal health	
Previous contraceptive method discontinuation due to unwanted side effects	992 (47.1)
Previous contraceptive method discontinuation due to unwanted changes in menses	516 (24.5)
History of menstrual problems (very heavy bleeding, irregular periods, or spotting between periods)	1,675 (79.6)
History of bacterial vaginosis	
** **Once or twice	799 (38.0)
** **Several/many times	223 (10.6)
Importance of nonhormonal contraception	
** **Not important	273 (13.0)
** **Somewhat important	553 (26.3)
** **Important	533 (25.3)
** **Very important	746 (35.4)
Experience with vaginal products	
Ever used a contraceptive vaginal ring	366 (17.4)
Knows others who have used a vaginal ring	701 (33.3)
Ever used other vaginal products^a^	1,981 (94.1)
HIV and STIs	
Perceived HIV risk (“How worried are you about getting HIV in the next 12 months?”)	
** **Not at all worried	1,551 (73.7)
** **Somewhat worried	365 (17.3)
** **Very worried	162 (7.7)
** **I am already living with HIV	8 (0.4)
** **Prefer not to answer	19 (0.9)
Perceived STI risk (for chlamydia, gonorrhea, herpes, warts, HPV, and/or syphilis)^b^	767 (36.4)
Sought care for suspected STI in the last year	386 (19.1)
Tested positive for at least one STI in the last year	186 (8.8)
Multiple (2 or more) male sexual partners in last year	553 (26.2)
MPT interest “If a contraceptive product (other than condoms) could also prevent HIV and other STIs, would you be more likely to use it?”	
** **No, not more likely	178 (8.5)
** **Yes, somewhat more likely	722 (34.3)
** **Yes, much more likely	999 (47.5)
** **Don't know	206 (9.8)

^a^ Included tampons, dildo or sex toys inserted into the vagina, sexual lubricant, vaginal medication, douche, or menstrual cup or disc.

^b^ “How worried are you about getting the following STIs in the next 12 months?” With response categories for each: “Not at all worried”, “Somewhat worried”, “Very worried”, “I currently have this STI”, or “Prefer not to respond”.

Of the 25% reporting worry about getting HIV in the next 12 months, 8% said they were “very worried” and 17% were “somewhat worried”. Eight women reported currently living with HIV. When asked about their worry about getting each of six different STIs in the next 12 months, 36% reported being at least “somewhat worried” about at least one of these STIs. 19% said they had sought care for a suspected STI in the last year, and 9% tested positive for at least one STI in that timeframe. One-quarter (26%) of women reported having multiple male sexual partners in the last year. Finally, when asked whether they would be more likely to use a contraceptive product (other than condoms) if it could also prevent HIV and other STIs (i.e., interested in an MPT), 48% said “Yes, much more likely”, and 34% said “Yes, somewhat more likely”.

### DCE results

The DCE was well-understood by participants, with 90% reporting that the choice sets were “somewhat easy” or “very easy” to complete, 9% “somewhat difficult” and 0.6% “very difficult”. When those saying somewhat/very difficult were asked what was difficult, 77% selected “Making choices between Option A, B, or neither” and 32% selected “Understanding the descriptions of the product characteristics (before I started making the choices)”.

#### What are women's preferences regarding each product attribute?

The mixed multinomial logit model (Table 3) yielded mean preference weights for each attribute. On average, women preferred higher prevention effectiveness for pregnancy, HIV, and STIs (each *p* < 0.001, with preference for pregnancy prevention about twice as high as that for HIV, which in turn was about twice as high as for STIs. The values of these coefficients represent preference for a 1% increase in effectiveness and, while comparable to each other, are not comparable with the other attributes. Women also preferred the product to prevent BV (*p* < 0.001), and to a much lesser extent, for the ring to be nonhormonal (vs. hormonal, *p* = 0.03). They had a negative preference for “may cause heavier/unpredictable periods” (vs. no effect on menses, *p* < 0.001). There was no preference for using the ring on-demand vs. only using it continuously.

**Table 3:** Mean preference weights from mixed multinomial logit model.

Attribute	Attribute levels	Coefficient	95% CI Lower, Upper		*p*- value	SD	SE of SD
Pregnancy prevention effectiveness	Continuous	0.021	0.017	0.024	<0.001	−0.012	0.002
HIV prevention effectiveness	Continuous	0.012	0.010	0.013	<0.001	0.013	0.001
STI prevention effectiveness	Continuous	0.006	0.005	0.007	<0.001	0.010	0.001
Prevents bacterial vaginosis	Prevents BV (Ref: Does not prevent BV)	0.305	0.264	0.346	<0.001	0.303	0.044
Effect on menses	May cause heavy/unpredictable bleeding (Ref: No effect on menses)	−0.440	−0.490	−0.389	<0.001	0.513	0.039
Nonhormonal	Nonhormonal (Ref: Hormonal)	0.047	0.089	0.004	0.032	0.467	0.037
Frequency of use	Can use continually or on-demand (Ref: Continuously use for one month)	−0.004	−0.039	0.031	0.826	0.034	0.039
ASC	Alternative specific constant for the opt-out	−0.374	−0.526	−0.222	<0.001	0.868	0.065

*n* = 75,780 observations. SD, standard deviation; SE, standard error.

#### How important are the attributes in relation to each other?

**Figure 3:**
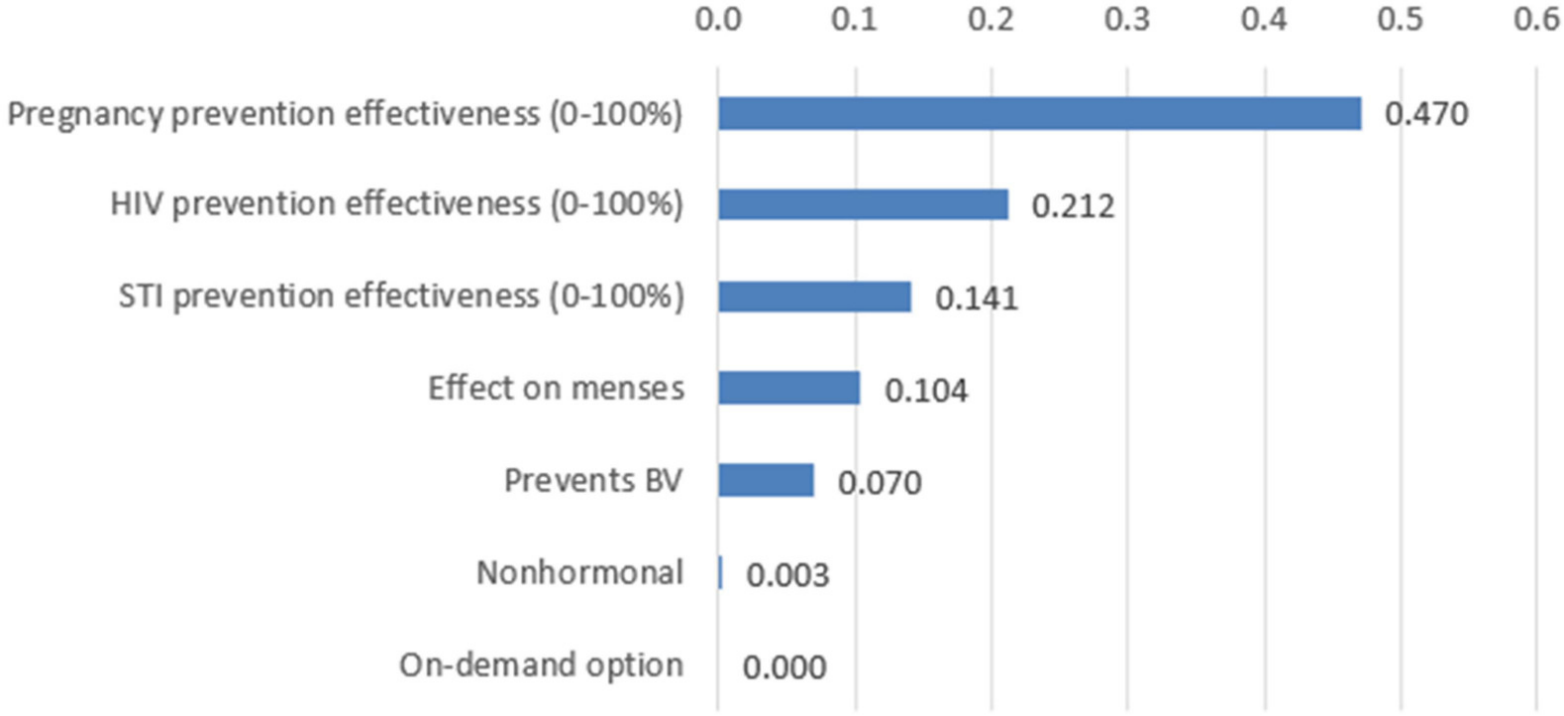
Relative importance of attributes based on DCE data (*n* = 2,105). Relative importance ranking calculations were sensitive to the original ranges of effectiveness for pregnancy, HIV, and STI prevention that participants were asked to consider within DCE choice sets. For improved comparability, this figure shows relative importance when effectiveness is considered on a scale from 0%–100%. Relative importance when considering the actual ranges of effectiveness women were asked to consider is included in Supplemental File 2.

Figure 3 shows the relative importance of attributes. Effectiveness for pregnancy prevention was the most important; effectiveness for HIV prevention was about half that of pregnancy prevention, and STI prevention about half that of HIV prevention. The importance of effect on menses and BV prevention were lower but still substantial, whereas relative importance of presence of hormones and frequency of use were negligible.

#### What level of pregnancy, HIV, and STI prevention effectiveness would women give up in order to get another preferred attribute?

Table 4 shows tradeoffs women would be willing to make in effectiveness for each indication (prevention of pregnancy, HIV, and STIs—the models at the top of the table) for each other attribute (listed along the left-hand column). For a 10% increase in pregnancy prevention effectiveness (see left-hand column), women would be willing to give up 25% HIV prevention effectiveness and 32% STI prevention effectiveness (both *p* < 0.001) (readers are reminded that these figures represent the coefficients, which reflect a 1% increase, multiplied by 10). For a 10% increase in HIV prevention effectiveness, women would be willing to give up 6% pregnancy prevention effectiveness and 2% STI prevention effectiveness (both *p* < 0.001). For a 10% increase in STI prevention effectiveness, they would be willing to give up 3% pregnancy prevention effectiveness and 7% HIV prevention effectiveness.

**Table 4:** Marginal rate of substitution for effectiveness of pregnancy, HIV, STI prevention, for each other attribute (*n* = 2,105).

Attribute	Attribute levels	WTP model for effectiveness of Pregnancy prevention				WTP model for effectiveness of HIV prevention				WTP for effectiveness of STI prevention			
		Coeff-icient	95% CI		*P* value	Coeff-icient	95% CI		*P* value	Coeff-icient	95% CI		*P* value
			Lower	Upper			Lower	Upper			Lower	Upper	
Pregnancy prevention effectiveness	Continuous *SD*	–	–	–	–	2.529 –*1.595*	1.980 –*1.891*	3.078 –*1.299*	<0.001	3.191 –*2.980*	2.700 –*3.470*	3.682 –*2.490*	<0.001
HIV prevention effectiveness	Continuous *SD*	0.596 *0.108*	0.467 *−0.011*	0.725 –*0.227*	<0.001	–	–	–	–	1.556 *–1.643*	1.327 *–1.910*	1.784 *–1.377*	<0.001
STI prevention effectiveness	Continuous *SD*	0.298 –*0.039*	0.247 *–0.098*	0.349 –*0.175*	<0.001	0.707 –*0.997*	0.577 *–1.120*	0.838 *–0.874*	0.000	–	–	–	–
Prevents bacterial vaginosis	Preventions BV (Ref: Does not prevent BV) *SD*	16.750 –*0.990*	12.831 –*1.335*	20.670 –*3.316*	<0.001	27.130 –*22.054*	23.627 –*29.364*	30.633 –*14.744*	0.000	38.626 –*24.445*	31.635 –*15.431*	45.617 –*33.459*	<0.001
Effect on menses	May cause heavy/unpredictable bleeding (Ref: No effect on menses) *SD*	−22.18 *0.817*	−26.06 *−2.029*	−18.305 –*3.663*	<0.001	−45.584 *−42.247*	−51.662 *−43.910*	−39.507 −*40.583*	0.000	−61.744 *−74.126*	−70.019 *−53.469*	−53.469 *−84.315*	<0.001
Non-hormonal	Nonhormonal (Ref: Hormonal) *SD*	1.461 *–0.400*	−0.627 *−2.914*	3.548 –*2.115*	0.170	6.096 *–37.410*	1.905 *–47.613*	10.287 *–27.208*	0.004	1.905 *–57.164*	−4.166 −*67.689*	7.975 –*46.640*	0.539
Frequency of use	Can use continually or on-demand (Ref: Continuously use for one month) *SD*	−1.309 *1.590*	−3.205 *−0.641*	0.586 –*3.821*	0.176	4.615 –*1.624*	1.399 –*5.437*	7.832 –*8.685*	0.005	−7.769 *−5.109*	3.808 *–12.050*	11.731 –*1.832*	<0.001
Model fit statistics													
Log-likelihood (final)		21,663.221				20,876.315				−20,922.415			
Number of Observations		75,780				75,780				75,780			
Number of Haltons		1,000				1,000				1,000			

Italicized values represent standard deviations (SD).

For the product to have no effect on menses (vs. may cause heavy/unpredictable bleeding) women would be willing to give up 22% pregnancy prevention effectiveness, 46% HIV, and 62% STI prevention effectiveness (all *p* < 0.001). For the ring to prevent BV, women would trade off 17% pregnancy prevention effectiveness, and 27% and 39% HIV and STI prevention effectiveness, respectively (all *p* < 0.001). Finally, women would not be willing to give up effectiveness for each indication (or only very minimal % effectiveness), for the ring to be nonhormonal or to be able to use the ring on-demand (vs. only continuously).

#### Do preferences for each attribute differ between respondents with different characteristics?

**Figure 4:**
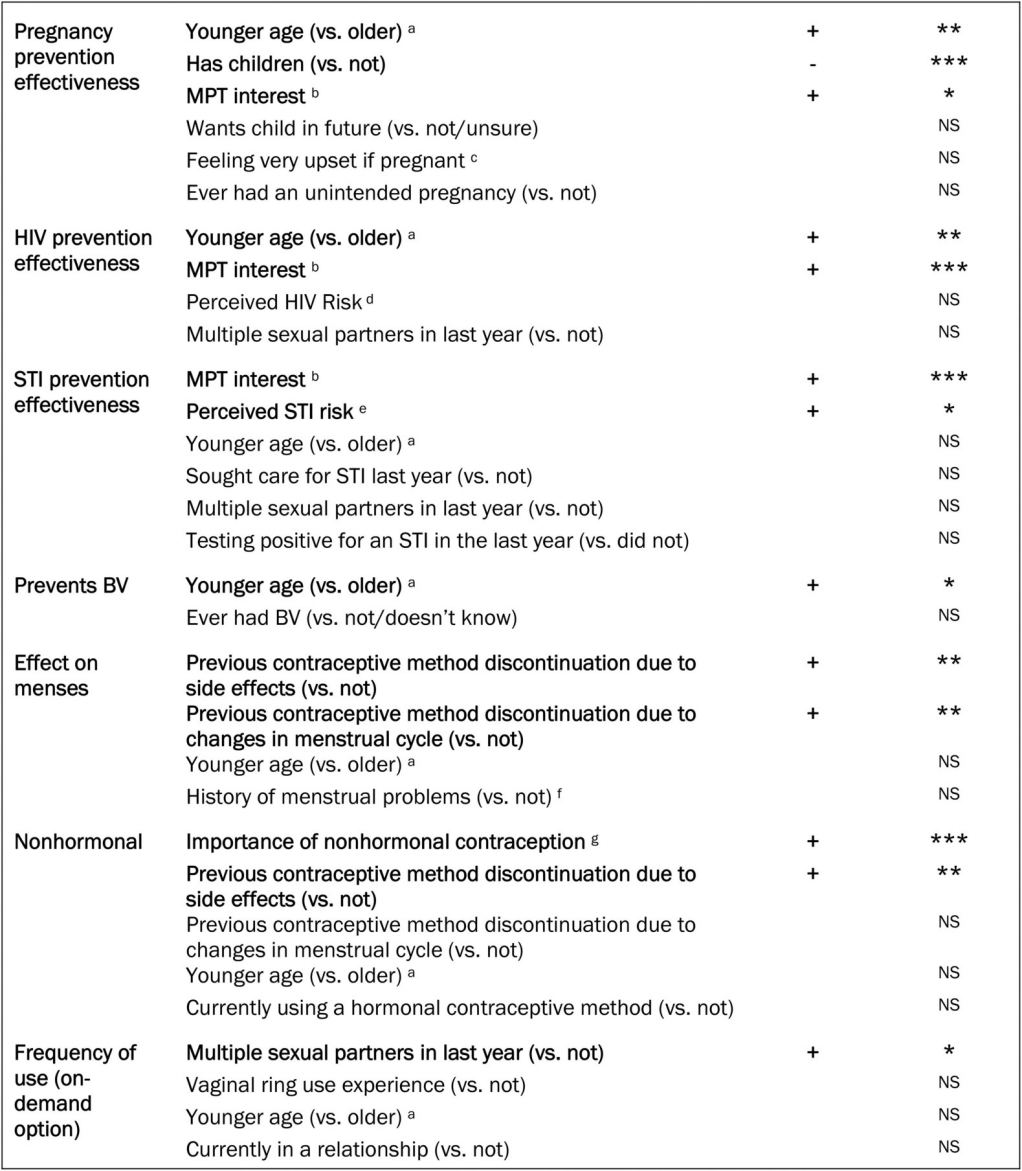
Summary of findings—respondent characteristics associated with preferences for each attribute. +Positive association—negative association *p < 0.05 **p < 0.01 ***p < 0.001 NS, not statistically significant. (a) 18–29 years (vs. 30–49). (b) Very likely to use MPT (vs. not likely/somewhat likely/don't know). (c) Very upset if found out pregnant (vs. somewhat upset, not upset, or unsure). (d) Somewhat/very worried about getting HIV (vs. not worried/has HIV/nonresponse). (e) Somewhat/very worried about getting at least one STI (chlamydia, gonorrhea, herpes, warts, HPV, syphilis) (vs. not worried). (f) Previous experience of menstrual problems (heavy bleeding, spotting, irregular, and/or no period) (vs. not). (g) Hormonal contraception is important/very important (vs. not important/somewhat important).

Results from the multinomial logit models that included interaction terms are described below and summarized in Figure 4, with more detailed results included in Supplementary Table 3. Results from selected subsample analyses are also described below.

##### Pregnancy prevention

There was a higher preference for pregnancy prevention effectiveness among younger women (ages 18–29 vs. 30–49 years, *p* < 0.01 for the interaction term). The subgroup of younger women preferred pregnancy prevention effectiveness about twice as much as older women (respective coefficients: 0.028 and 0.014, both *p* < 0.001 for difference with zero). Women with no children (vs. ≥1 child) also had a higher preference for pregnancy prevention effectiveness (*p* < 0.001 for the interaction term), as did women who said they would be “very likely” to use an MPT in general (*p* < 0.01 for the interaction term).

##### HIV prevention

There was also a higher preference for HIV prevention effectiveness among younger women (ages 18–29 vs. 30–49, *p* = 0.001 for interaction term). Preference for HIV prevention effectiveness did not significantly differ between women worried about HIV vs. not (i.e., the interaction term was non-significant). However, among the subgroup of women worried about HIV, preference for HIV prevention effectiveness was much higher, similar to that of pregnancy prevention effectiveness (respective coefficients, 0.011 and 0.009; *p*-values for differences with zero, *p* < 0.001 and *p* < 0.01). Finally, women who are very interested in an MPT had higher preference for HIV prevention effectiveness (*p* < 0.001 for the interaction term); this subgroup would accept a 10.7% reduction in pregnancy prevention effectiveness for a 10% increase in HIV prevention effectiveness (95% CI 6.2%, 15.3%; *p* < 0.001).

##### STI prevention

There was a higher preference for STI prevention effectiveness among women worried about getting an STI in the next year vs. not worried (*p* < 0.05 for the interaction term). The subgroup of women worried about getting an STI preferred STI prevention effectiveness about twice as much as those not worried (respective coefficients: 0.007; 0.003). Women worried about STIs would be willing to accept a 4.6% reduction in pregnancy prevention effectiveness for a 10% increase in STI prevention effectiveness (95% CI 2.6%, 6.5%, *p* < 0.001). Women who are very interested in an MPT also had a higher preference for STI prevention effectiveness (*p* < 0.001 for the interaction term), and would accept a 5.4% reduction in pregnancy prevention effectiveness for a 10% increase in STI prevention effectiveness (95% CI 3.6%, 7.2%; *p* < 0.001). There was no difference in preference for STI prevention effectiveness between women with and without multiple sexual partners in the last year, or who sought care for an STI in the last year (vs. did not seek care).

##### BV prevention

BV prevention was preferred more among younger women (ages 18–29 vs. 30–49; *p* < 0.05 for interaction term; respective subgroup coefficients 0.335 and 0.264; both *p* < 0.001 for differences with zero). There was no difference in BV prevention interest based on BV history.

##### Impact on menses

Women who had discontinued previous methods due to effects on menses (vs. not) had over 1.5 times the negative preference (respective coefficients: −0.550, −0.258) for the ring to potentially cause heavier or unpredictable bleeding (vs. no effect) (*p* < 0.01 for interaction term). These women would be willing to accept a 29.8% decrease in pregnancy prevention effectiveness for the ring to have no effect on menses (95% CI 21.2%, 38.0%; *p* < 0.001). There was no difference in preferences regarding the ring's effect on menses between women with and without a history of menstrual problems.

##### Hormonal vs. nonhormonal

There was a higher preference for the ring to be nonhormonal among women for whom nonhormonal contraception is important (*p* < 0.001 for the interaction term; coefficient of 0.130 is significantly greater than zero, at *p* < 0.001). This group of women would be willing to accept a 14.2% reduction in pregnancy prevention effectiveness for the ring to be nonhormonal (95% CI 7.1%, 21.6%; *p* < 0.001). Women who previously discontinued a contraceptive due to unwanted side effects also preferred the ring to be nonhormonal (*p* < 0.01 for the interaction term).

##### On-demand vs. continuous use

Finally, there was a modestly higher preference for being able to use the ring on-demand (vs. only continuously) among women with multiple sexual partners (*p* < 0.05 for the interaction term). However, among the subgroup of women with multiple partners, there was no significant preference for an on-demand option for the ring, nor willingness to accept any reduction in pregnancy, HIV, or STI prevention effectiveness for an on-demand option. Additionally, there was no difference in preference for on-demand or continuous use among those who had or had not previously used a vaginal ring.

#### Direct elicitation of likelihood of using the nonhormonal MPT ring product

**Figure 5:**
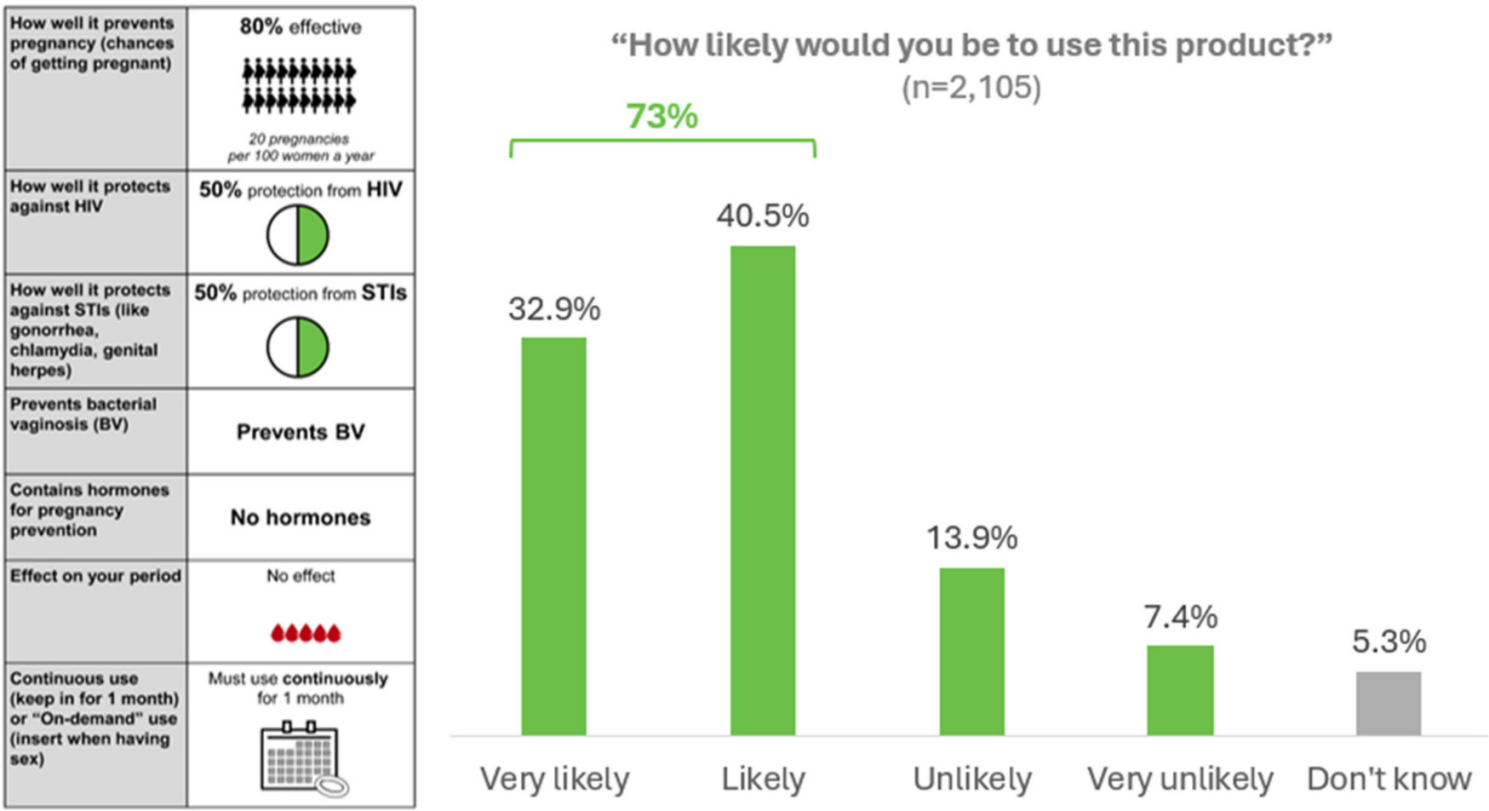
Likelihood of using MPT ring with the following product profile (asked after DCE choice sets).

As shown in Figure 5, when asked how likely they would be to use a nonhormonal MPT vaginal ring with conservative effectiveness levels and that prevents BV, has no effect on menses, and must be worn continuously, nearly three-quarters of women said they would be likely to use it (33% “very likely” and 40% “likely”). Of those, nearly 70% said they would start using the ring within a year of it being approved (over half of those “right away”) (Table 5). About three-quarters (76%) would use it for both pregnancy and HIV/STI prevention, vs. for pregnancy or HIV/STI prevention alone. If given the choice, one-third (33%) would prefer using the ring continuously (for its 1-month duration); 31% at each sex act; 36% intermittently (a few days/weeks at a time). Nearly three-quarters (73%) would remove it during menses. Most women (83%) were fine with changing to a new ring each month.

**Table 5:** Anticipated MPT ring use practices, and reasons for being unlikely to use the product**.**

Anticipated use practices for the MPT ring (among women likely/very likely to use the MPT ring as described)	*n* = 1,545 *n* (%)
How soon after the method is approved by the FDA, would you want to start using it?	
** **Right away	591 (38.3)
** **In 6–12 months	472 (30.6)
** **In 1–2 years	308 (19.9
** **In 3 + years^a^	142 (9.2)
Indications (select one)	
** **Pregnancy prevention only	222 (14.4)
** **HIV/STI prevention only	142 (9.2)
** **Both pregnancy and HIV/STI prevention	1,181 (76.4)
How they would want to use the ring (select one)^b^	
** **Use it at each sex act (like a condom)	480 (31.1)
** **Use it to cover a few days at a time when you know you'll be having sex, but remove it otherwise	375 (24.3)
** **Use it a week or two at a time when you know you'll be having sex, but remove it otherwise	190 (12.3)
** **Use it continuously	500 (32.6)
Would be ok with replacing the vaginal ring with a new one each month (agree/strongly agree)	1,282 (83.0)
Reasons for being unlikely to use the product (among women unlikely/very unlikely to use the MPT ring as described) (can select multiple)	***n*** **=** **449 *n* (%)**
** **I am happy with my current method(s)	248 (55.2)
** **I would want higher effectiveness for pregnancy prevention	187 (41.7)
** **I would want higher effectiveness for HIV or STI prevention	96 (21.4)
** **I’m not comfortable with the idea of using a vaginal ring	121 (27.0)
** **I wouldn't want to wear a ring for a whole month	92 (20.5)
** **I don't understand/don't trust the active ingredients in this ring	34 (7.6)
Other	14 (3.1)

^a^ An additional 32 participants chose “prefer not to respond”.

^b^ The full wording for this survey question was “It is possible the vaginal ring we are developing could be used continuously OR on-demand (only when you have sex). If so how do you think you would want to use the ring?”.

Among women saying they would be unlikely to use the MPT ring (*n* = 449), the main reasons included happiness with their current method(s) (55%), wanting higher pregnancy prevention effectiveness (42%) or HIV/STI prevention effectiveness (21%), and/or being uncomfortable with the idea of using a vaginal ring (27%).

We also conducted exploratory ordinal logistic regression analyses (Supplementary Table 4) to identify characteristics of women reporting greater likelihood of using the nonhormonal MPT-ring as described. In the final adjusted model (achieved using backward elimination), higher likelihood of using the ring was significantly associated with (in order starting with highest adjusted odds ratio): experience using a vaginal ring (aOR 1.96, *p* < 0.001), strong preference for nonhormonal contraception (aOR 1.71, *p* < 0.001), having child(ren) (aOR 1.69, *p* < 0.001), HIV worry (aOR 1.39, *p* < 0.01), knowing others who have used a vaginal ring (aOR 1.36, *p* < 0.01), STI worry (aOR 1.28, *p* < 0.05), seeking care for an STI in the last year (aOR 1.26, *p* < 0.05), history of heavy menses (aOR 1.21, *p* < 0.01), and urban residence (vs. suburban or rural) (aOR 1.15, *p* < 0.05).

## Discussion

Among the diverse sample of 2,105 US women completing the online survey, nearly three-quarters (74%) expressed interest in using a nonhormonal MPT vaginal ring. Effectiveness for pregnancy and HIV prevention were the most important MPT ring attributes, but STI and BV prevention were also valued. Preferences were somewhat heterogeneous, especially for HIV prevention effectiveness (more important to younger women and those worried about HIV) and a nonhormonal option (only important to women desiring nonhormonal contraception and with previous unwanted effects from hormonal methods). Women were also willing to make tradeoffs in pregnancy, HIV, and STI prevention effectiveness, for other desired ring attributes.

The SRH histories and experiences of the women we surveyed underline the need for more acceptable prevention product options (58). Reports of unintended pregnancy were prevalent (43%) and similar to the 42% found in another recent national survey (59). Contraceptive method discontinuation due to unwanted effects was also prevalent, and the most common currently used contraceptives (male condoms, birth control pills, withdrawal, and fertile days), are of mid- or low-tier effectiveness (60). Moreover, a substantial proportion of women (9%) were diagnosed with an STI in the last year, and while one-quarter of the participants perceived themselves to be at risk for HIV, only 5% were using highly effective biomedical prevention [these findings are described in more detail elsewhere (61)].

DCE findings showed that pregnancy prevention was about twice as important as HIV prevention, which in turn was about twice as important as STI prevention. However, among women worried about getting HIV, HIV prevention was as important as pregnancy prevention. Similar preferences have also been found in several other studies, although these studies were conducted 8–10 years ago. In a US national survey, sexually active women ages 18–29 years were asked about hypothetical MPT product features and ranked the importance of effectiveness for pregnancy prevention only marginally higher than for HIV prevention (40). Another study in South Africa found that importance of HIV prevention effectiveness equaled or exceeded that for pregnancy, particularly among subgroups of women at highest risk of HIV (62). That STI prevention effectiveness, while still important, was not as important as HIV prevention, may reflect women's awareness of how common STIs are, and greater perceived severity of HIV than STIs (51). Finally, women also valued BV prevention—with younger women preferring it more than older women. Interestingly, however, the 49% of women with a history of BV did not prefer a ring that prevents BV more than those without.

Overall, participants did not exhibit a general preference between nonhormonal and hormonal contraception. However, the 35% of women who considered nonhormonal contraception very important were willing to accept up to a 14% reduction in pregnancy prevention effectiveness for a nonhormonal option. Similarly, women who had previously discontinued contraceptive use due to unwanted side effects—often associated with hormonal methods—also showed a marked preference for nonhormonal contraception.

There was also a strong negative preference for “may cause heavy/unpredictable bleeding”, vs. “no effect on menses”, especially women who reported discontinuing previous contraceptive methods for this reason. Of note, comparing heavier/unpredictable bleeding with “no effect” may be somewhat difficult because while most women would not want heavier/unpredictable bleeding, some women may actually want the ring to effect on menses, such as by causing lighter menses or no menses (63). Given individual variability in menstrual bleeding experiences, understanding the impact of unpredictable bleeding and determining ways to mediate this in the future will be important for contraceptive satisfaction.

There was no preference for an on-demand option (vs. continuous use only) in the DCE, although there was a marginally significant preference among women with multiple sexual partners in the last year (which could indicate more intermittent periods of risk, lessening the perceived need for continuous use). When asked directly, after the DCE, how they would want to use the product, two-thirds said on-demand or intermittently, and only one-third continuously. It may be that the strength of the other attributes in the DCE model obscured an effect for an on-demand option. Whether an MPT ring needs to be used continuously or can be used on-demand may not be a predominant consideration for most women, even though preferences do appear to exist.

Many women (73%) reported being likely to use the nonhormonal MPT ring product, even with quite conservative levels of effectiveness (which are anticipated to be higher in the eventual product). The authenticity of this stated interest was reinforced by the fact that nearly 70% said they would want to start using it within the first year after it was approved by the FDA [often used as an indicator of true interest in preference research about hypothetical products, including contraception (28, 31, 64)]. The high interest may speak to the dearth of alternative acceptable options for contraception and/or HIV/STI prevention (14, 58). It also may suggest a degree of comfort with the idea of vaginal rings, perhaps due to a growing familiarity with rings. Many women in our sample were familiar with rings, with 17% ever using a ring and 33% knowing others who have. Moreover, the variable most strongly associated with increasing likelihood of using the MPT ring was ever use of a contraceptive vaginal ring (with knowing others who used one also significantly associated), suggesting that product uptake may happen first among women already familiar with rings. Previous preference research among women that has compared potential types of MPTs, including in the US, has tended to find vaginal rings are somewhat less preferred than vaginal gels, pills, or injectables (9, 12, 40, 62), perhaps in part because the latter methods were more familiar to participants.

Additionally, most women said they would use the product for both pregnancy and HIV/STI prevention, vs. for one or the other—as also reflected in the high stated interest in MPTs in general—whereas only one-quarter of women reported being worried about HIV, and 36% about at least one STI that we asked about. Other studies have also shown that a large majority of women would prefer a product with multiple vs. a single indication (10–12, 39, 65). It may be that women who are not necessarily worried about HIV/STIs still perceive an advantage of a product that offers to prevent them in addition to women's primary concern of preventing pregnancy.

This study had several limitations. First, the study is based on hypothetical willingness to use a novel product, which may introduce uncertainty or bias in responses. As there are no existing MPT rings on the market, we can only use a hypothetical approach to help inform product development efforts and ensure alignment with end-user needs. Second, we recognize that the theoretical estimands in this study, i.e., (relative) MPT ring attribute preferences and overall acceptability, as well as heterogeneity in each, can only be approximated by the selected empirical estimands (47) from the DCE, interaction, and regression models, each of which is also constrained by its own assumptions. Third, even with visuals and pretesting, a non-trivial share of respondents found the DCE tasks to be somewhat (9%) or very (1%) difficult, which may have added statistical noise/random variation to the data. Fourth, we recognize that the selection of attribute levels may not have fully captured potential choices; in particular, while for the effect on menses attribute, the level “heavier/more unpredictable bleeding” (vs. no effect) was chosen because those are the most likely results of copper in the nonhormonal MPT ring, it may have been preferable to separate “heavier” from “unpredictable” effects, which future research should do. Fifth, the different range of levels of the continuous effectiveness attributes reduced direct comparability of their relative importance. Finally, the sample may not be fully representative of sexually active US women ages 18–49 years who currently/are interested in using contraception, and with no tubal ligation/hysterectomy (i.e., our study's eligibility criteria). Due to our purposive oversampling of Black/African American women, the sample certainly over-represents that subgroup. However, we achieved a large, diverse sample with prevalent SRH needs. Of note, comparable available national socio-demographic data for US women of reproductive age suggest that educational attainment among women in our survey was similar to that of women nationally (e.g., about half had completed high school, about one-third had an Associates/Bachelor's degree, and about 10% a graduate degree) (66), and about half of women had at least one biological child (67).

Findings from this study, among a large, diverse sample of US women, suggest substantial interest in a nonhormonal MPT ring, even one with more conservative estimates of effectiveness. Effectiveness for pregnancy and HIV prevention emerged as most important, but STI and BV prevention were also valued. Preferences were somewhat heterogeneous, with desired levels of prevention effectiveness and nonhormonal options in particular differing according to specific characteristics of women. Such preference heterogeneity should be considered in product design and suggests a need for individualized counseling. Continued end-user preference research is needed in varied settings around the world to optimize the development and introduction of future MPT products, including vaginal rings.

## Data Availability

The raw data supporting the conclusions of this article will be made available by the authors, without undue reservation.
