# Supplementary material for: Multipurpose vaginal rings: preferences from a national discrete choice experiment survey among US women
Source: Front Reprod Health. 2026 Feb 19;8:1722593. doi: 10.3389/frph.2026.1722593 (PMC12960174; doi:10.3389/frph.2026.1722593)
Supplement: Supplementary file 1 [file Supplementaryfile1.docx]

**SUPPLEMENTAL FILES**

**Supplemental File 1. DCE design**

**
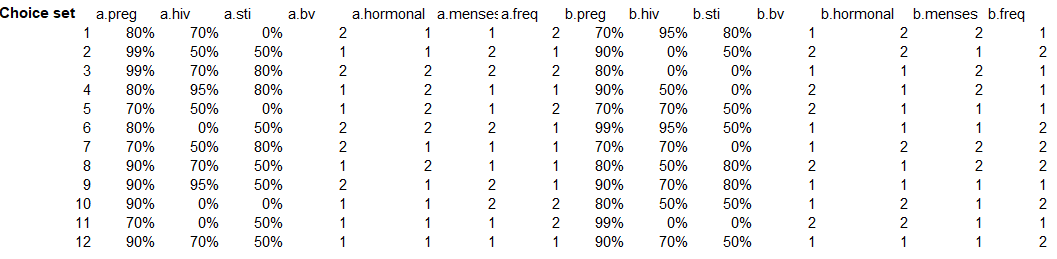
**

**
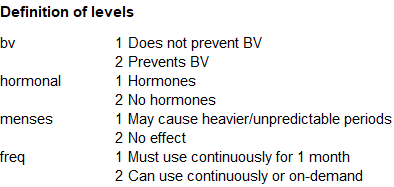
**

**Supplemental File 2. Text introducing and describing the DCE choice sets**


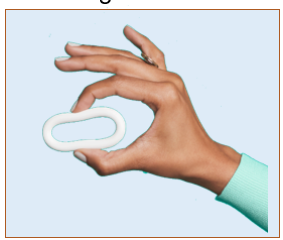
The DCE section was introduced by the following text (next to the picture to the right, of a hand gently squeezing an off-white-colored vaginal ring): “We are developing a new method for women. It is a ring that a woman would wear inside her vagina, of similar size as other vaginal rings already available. The ring we’re developing will help prevent pregnancy, and may also help prevent STIs and/or HIV. This product is still being developed, and we want to make sure its characteristics meet the needs and preferences of as many women as possible. We will ask your opinions and there are no right or wrong answers.”

We then asked women to view a six-second-long video graphic of ring insertion and placement. Next, we asked women to view a series of informational statements, to which she had to click “Ok, got it.” to move onto the next. These statements included:

- “A woman inserts the ring into her vagina, and removes it, herself (rather than a health care provider doing it). Most women using vaginal rings say they do not feel them during daily activities or during sex.”
- “There are currently two vaginal rings to prevent pregnancy that are approved by the FDA and are available by prescription. They are called NuvaRing® and Annovera®. Both of these contain hormones.”
- “The ring we are developing needs to remain in the vagina during sex to be effective.”
- “Each ring will last about a month and then needs to be replaced with a new ring.
- “You can either keep it in or take it out during your period, although you’ll need to keep it in if you have sex during your menstrual period. You can use tampons while wearing the ring.”

We then reviewed the seven attributes of the ring women would be asked about (i.e., as attributes in the DCE choice sets), for which they needed to click “Ok, got it.” These descriptions included:

-
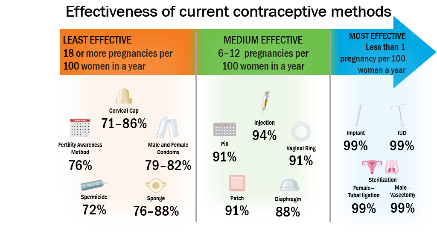
How well it prevents pregnancy is how well the ring works to prevent pregnancy. Options are 70%; 80%, 90%, and 99%. The graphic below shows how effective currently available pregnancy prevention options are.  For example, the effectiveness level for NuvaRing is 91%, and some common side effects include headaches, nausea, and changes in menses (your period). Fertility awareness is 76% effective and has no side effects.
- How well it protects against HIV is how well the ring works to prevent HIV. Options are 0% (does not prevent HIV), 50%, 70%, and 95%.
- How well it protects against HIV is how well the ring works to prevent HIV. Options are 0% (does not prevent HIV), 50%, 70%, and 95%.
- How well it protects against STIs is how well the ring works to prevent STIs such as chlamydia, gonorrhea, and genital herpes. Options are 0% (does not prevent STIs); 50%; and 80%. Note that aside from condoms, there are no other products available to prevent these STIs.
- Prevents bacterial vaginosis (BV) means whether or not the ring can prevent BV. BV can cause abnormal discharge, itching or odor.
- Contains hormones means whether or not the ring contains hormones in order to prevent pregnancy. For a ring that does NOT contain hormones, the active ingredients could be copper or zinc, which have been used in contraceptives before. These ingredients will prevent sperm from fertilizing an egg.
- Effect on your period is whether the ring may cause heavier and/or unpredictable periods, or has no effect. Please assume any effect would occur for as long as the ring is used.
- Continuous use or on-demand use means whether the ring must be used continuously for one month, VERSUS whether it can either be used continuously OR “on-demand”. “On-demand” means using the ring for each sex act (like a condom) or for a few days at a time when you know you'll be having sex, but removing it otherwise.

Women were also told: “When answering questions about the ring, please assume that availability and cost will not be barriers to using it.” A final statement before proceeding to the 12 DCE choice sets was: “We will now ask you to make a series of choices between two hypothetical vaginal ring options – Option A or Option B. Please take your time to consider the two options and choose Option A, Option B, or neither.”

**Supplemental File 3. Correlation matrix for variables that were included in the MMNL interaction models and/or the multivariate ordinal logistic regression analyses (Supplemental Tables 3 and 4, respectively)**

**
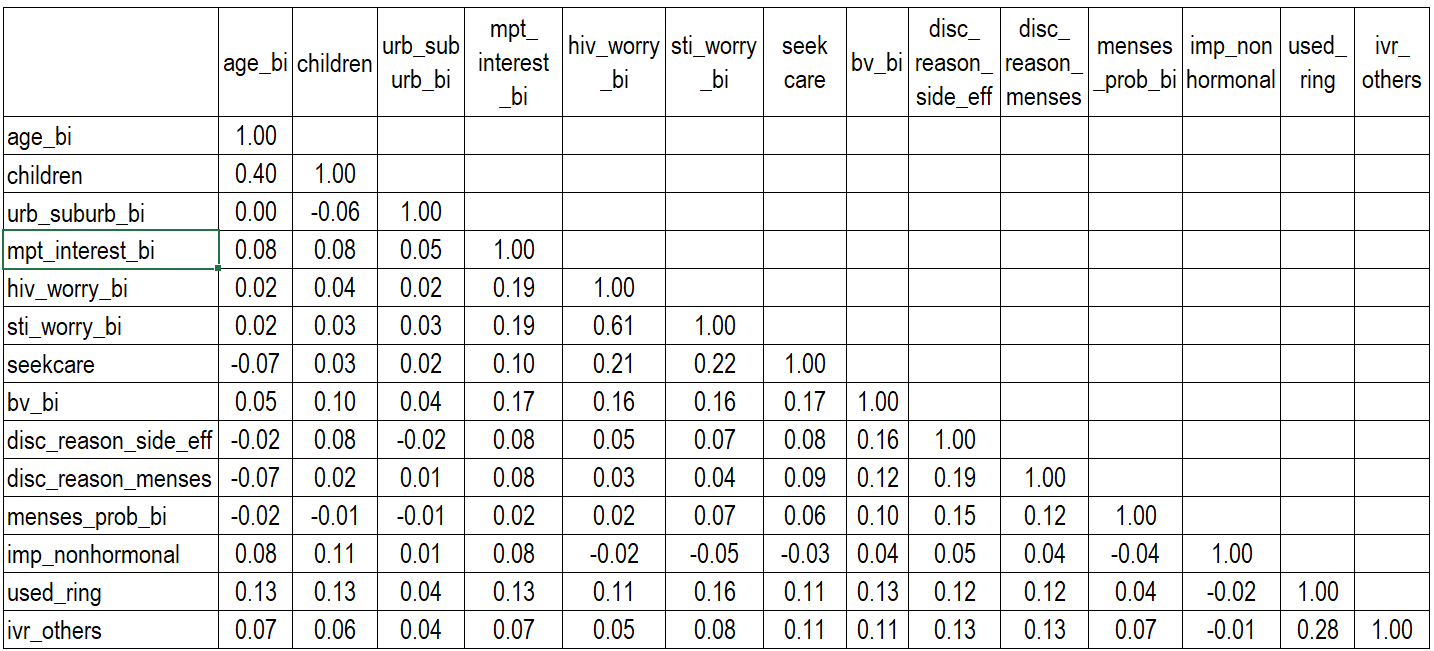
**

**Supplemental Table 1. Contraceptive and HIV prevention product use history (n=2,105)**

| **Contraceptive use history**  (in order of current use prevalence) | **Currently using**  n (%) | **Ever used**  (but not currently)  n (%) | **Never used**  n (%) |
| --- | --- | --- | --- |
| **Male condoms** | 898 (42.7) | 880 (41.8) | 327 (15.5) |
| **Birth control pills** | 669 (31.8) | 1,036 (49.2) | 400 (19.0) |
| **Withdrawal** | 560 (26.6) | 640 (30.4) | 905 (43.0) |
| **Fertile days** | 269 (12.8) | 356 (16.9) | 1,480 (70.3) |
| **Emergency contraception**  (e.g., Plan B, Ella levonorgestrel) | 209 (9.9) | 695 (33.0) | 1,201 (57.1) |
| **Contraceptive implant**  (Implanon, Nexplanon) | 192 (9.1) | 253 (12.0) | 1,660 (78.9) |
| **Contraceptive injection**  (Depo-Provera) | 187 (8.9) | 560 (26.6) | 1,358 (64.5) |
| **Hormonal IUD**  (Levonorgestrel IUD, Mirena, Liletta, Skyla) | 176 (8.4) | 249 (11.8) | 1,680 (79.8) |
| **Female condoms** | 140 (6.7) | 274 (13.0) | 1,691 (80.3) |
| **Vaginal ring** (NuvaRing, Annovera) | 83 (3.9) | 283 (13.4) | 1,739 (82.6) |
| **Spermicide** | 82 (3.9) | 236 (11.2) | 1,787 (84.9) |
| **Copper IUD** (Paraguard) | 65 (3.1) | 162 (7.7) | 1,878 (88.2) |
| **Birth control patch** | 64 (3.0) | 422 (20.1) | 1,619 (76.9) |
| **Male sterilization** (vasectomy) | 64 (3.0) | 117 (5.6) | 1,924 (91.4) |
| **Diaphragm** (with/without spermicide) | 48 (2.3) | 134 (6.4) | 1,923 (91.4) |
| **HIV prevention product use history** (in order of current use prevalence) | **Currently using**  n (%) | **Ever used**  (but not currently)  n (%) | **Never used**  n (%) |
| **Male condoms** | 1,011 (48.0) | 878 (41.7) | 216 (10.3) |
| **Pre-exposure prophylaxis (PrEP)** (oral or injectable) | 95 (4.5) | 175 (8.3) | 1,835 (87.2) |
| **Female condoms** | 65 (3.1) | 150 (7.1) | 1,630 (77.4) |
| **Post-exposure prophylaxis (PEP)** | 39 (1.9) | 103 (4.9) | 1,963 (93.3) |

**Supplemental Table 2. Mean preference weights from conditional logit model (estimated before the mixed multinomial logit model, which was the final model)**

|  |  | **Conditional Logit model** | | | |
| --- | --- | --- | --- | --- | --- |
| **ATTRIBUTE** | **Attribute levels** | **Coeff-icient** | **95 % CI**  **Lower, Upper** | | **p-value** |
| **Pregnancy prevention effectiveness** | Continuous | 0.016 | 0.137 | 0.018 | <0.001 |
| **HIV prevention effectiveness** | Continuous | 0.008 | 0.008 | 0.009 | <0.001 |
| **STI prevention effectiveness** | Continuous | 0.004 | 0.004 | 0.005 | <0.001 |
| **Prevents bacterial vaginosis** | Preventions BV (Ref: Does not prevent BV) | 0.257 | 0.223 | 0.290 | <0.001 |
| **Effect on menses** | May cause heavy/unpredictable bleeding (Ref: No effect on menses) | -0.335 | -0.368 | -0.301 | <0.001 |
| **Nonhormonal** | Nonhormonal (Ref: Hormonal) | 0.241 | -0.007 | 0.547 | 0.124 |
| **Frequency of use** | Can use continually or on-demand (Ref: Continuously use for one month) | -0.038 | -0.005 | -0.071 | <0.001 |
| **ASC** | Alternative specific constant for the opt-out | 0.107 | 0.006 | 0.208 | 0.037 |

**Supplemental file 4. Relative importance of attributes based on DCE data and when** **considering the actual range from the lowest to highest prevention efficacies women were asked to consider (70-99% for pregnancy, 0-95% for HIV, 0-80% for STIs)**

**
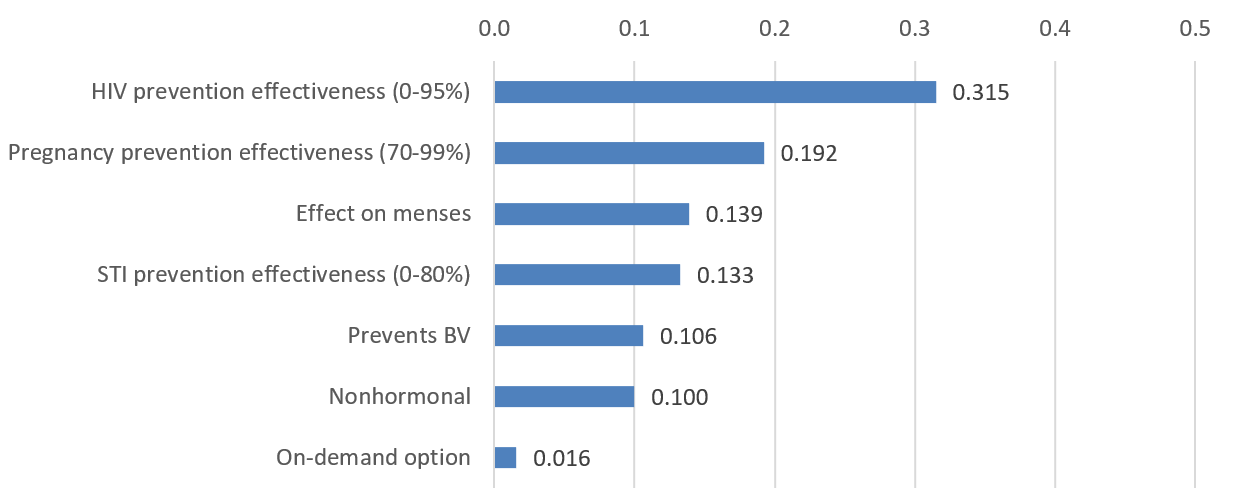
**

**Supplemental Table 3. Interactions between each attribute and selected participant characteristics (full sample)**

| **Participant characteristic** |  | **Coefficient for interaction term (MMNL model)** | **95% CI** | | **p-value** |  |  |
| --- | --- | --- | --- | --- | --- | --- | --- |
|  |  |  | **Lower** | **Upper** |  |  |  |
| **Interaction with pregnancy prevention effectiveness** [the following interaction terms were non-significant in the CLM model and thus were not entered into MML models: feeling very upset if pregnant, unintended pregnancy history] | | | | | | |  |
| Younger age | 18-29 years (vs. 30-49)  *SD*  *Main effect* | **0.005**  *-0.003*  *0.018* | **0.002**  *-0.007*  *0.015* | **0.007**  *0.000*  *0.022* | **0.003** |  |  |
| Has Children | One or more children (vs. no child)  *SD*  *Main effect* | **-0.007**  *0.000*  *0.024* | **-0.010**  *-0.002*  *0.020* | **-0.004**  *0.003*  *0.028* | **<0.001** |  |  |
| Whether wants child in future | Wants more children (vs. does not want child or not sure)  *SD*  *Main effect* | **0.001**  *0.004*  *0.020* | **-0.002**  *-0.013*  *0.016* | **0.004**  *0.021*  *0.023* | **0.419** |  |  |
| MPT Interest | Very likely to use MPT (vs. not likely/somewhat likely/don’t know)  *SD*  *Main effect* | **0.003**  *0.003*  *0.019* | **0.000**  *-0.002*  *0.016* | **0.006**  *0.007*  *0.023* | **0.042** |  |  |
| **Interaction with HIV prevention effectiveness** | | | | | | |  |
| Younger age | 18-29 years (vs. 30-49)  *SD*  *Main effect* | **0.003**  *0.004*  *0,010* | **0.001**  *0.000*  *0.009* | **0.005**  *0.008*  *0.011* | **0.001** |  |  |
| Perceived HIV Risk | Somewhat/very worried about getting HIV (vs. not worried/has HIV/nonresponse)  *SD*  *Main effect* | **0.001**  *0.009*  *0.011* | **-0.002**  *0.005*  *0.010* | **0.003**  *0.013*  *0.012* | **0.748** |  |  |
| Multiple sexual partners | Two or more partners in last 12 months (vs. 1 or none)  *SD*  *Main effect* | **0.001**  *0.005*  *0.010* | **-0.005**  *0.000*  *0.004* | **0.007**  *0.009*  *0.016* | **0.720** |  |  |
| MPT Interest | Very likely to use MPT (vs. not likely/somewhat likely/don’t know)  *SD*  *Main effect* | **0.006**  *0.007*  *0.009* | **0.004**  *0.001*  *0.008* | **0.007**  *0.013*  *0.010* | **<0.001** |  |  |
| **Interaction with STI prevention effectiveness**  [the following interaction terms were non-significant in the CLM model and thus were not entered into MML models: Testing positive for an STI in the last year] | | | | | | |  |
| Younger age | 18-29 years (vs. 30-49)  *SD*  *Main effect* | **0.001**  *0.005*  *0.006* | **0.000**  *0.001*  *0.005* | **0.003**  *0.009*  *0.007* | **0.187** |  |  |
| Perceived STI risk (for chlamydia, gonorrhea, herpes, warts, HPV, syphilis) | Somewhat/very worried about getting STIs  *SD*  *Main effect* | **0.002**  *0.001* | **0.000**  *-0.011* | **0.003**  *0.014* | **0.022** |  |  |
| MPT Interest | Very likely to use MPT (vs. not likely/somewhat likely/don’t know)  *SD*  *Main effect* | **0.006**  *0.005*  *0.003* | **0.004**  *0.002*  *0.002* | **0.007**  *0.009*  *0.004* | **<0.001** |  |  |
| Care seeking for STI in last year | Sought care for STI last year (vs. did not seek care/nonresponse)  *SD*  *Main effect* | **-0.001**  *0.000*  *0.006* | **-0.003**  *-0.001*  *0.005* | **0.001**  *0.584*  *0.007* | **0.361** |  |  |
| Multiple sexual partners | Two or more partners in last 12 months (vs. 1 or none)  *SD*  *Main effect* | **0.000**  *0.007*  *0.006* | **-0.004**  *0.000*  *0.002* | **0.004**  *0.014*  *0.010* | **0.962** |  |  |
| **Interaction with preventing bacterial vaginosis** | | | | | | |  |
| Younger age | 18-29 years (vs. 30-49)  *SD*  *Main effect* | **0.073**  *0.067*  *-0.269* | **0.007**  *-0.008*  *-0.323* | **0.140**  *0.142*  *-0.215* | **0.031** |  |  |
| History of BV | Ever had bacterial vaginosis (vs. no exposure/don’t know)  *SD*  *Main effect* | **0.013**  *-0.168*  -0.302 | **-0.054**  *0.043*  *-0.354* | **0.080**  *0.293*  *-0.250* | **0.710** |  |  |
| **Interaction with effect on menses** | | | | | | |  |
| Younger age | 18-29 years (vs. 30-49)  *SD*  *Main effect* | **0.047**  *0.161*  *-0.307* | **-0.029**  *-0.080*  *-0.348* | **0.122**  *0.402*  *-0.266* | **0.225** |  |  |
| Previous contraceptive method discontinuation due to side effects | Discontinued previous method due to side effects (nausea, headaches, weight gain, pain or discomfort) (vs. did not discontinue for these reasons)  *SD*  *Main effect* | **0.120**  *-0.315*  *-0.380* | **0.044**  *-0.460*  *-0.440* | **0.120**  *-0.169*  *-0.320* | **0.002** |  |  |
| Previous contraceptive method discontinuation due to changes in menstrual cycle | Discontinued previous method due to changes on menstrual cycle (vs. did not)  *SD*  *Main effect* | **0.141**  *0.213*  *-0.404* | **0.050**  *-0.128*  *-0.458* | **0.231**  *0.554*  *-0.350* | **0.002** |  |  |
| History of menstrual problems | Previous experience of menstrual problems (heavy bleeding, spotting, irregular, and/or no period) (vs. not)  *SD*  *Main effect* | **0.050**  *0.264*  *-0.396* | **-0.044**  *0.148*  *-0.486* | **0.145**  *0.380*  *-0.307* | **0.295** |  |  |
| **Interaction with nonhormonal**  [the following interaction terms were non-significant in the CLM model and thus were not entered into MML models: Age group; Currently using a hormonal contraceptive method] | | | | | | |  |
| Importance of nonhormonal contraception | Important /very important (vs. not important/somewhat important)  *SD*  *Main effect* | **0.271**  *0.556*  *-0.151* | **0.202**  *0.473*  *-0.201* | **0.340**  *0.639*  *-0.102* | **<0.001** |  |  |
| Previous contraceptive method discontinuation due to side effects | Discontinued previous method due to side effects (nausea, headaches, weight gain, pain or discomfort) (vs. did not discontinue for these reasons)  *SD*  *Main effect* | **0.096**  *0.280*  *0.008* | **0.024**  *0.075*  *-0.044* | **0.169**  *0.485*  *0.059* | **0.009** |  |  |
| Previous contraceptive method discontinuation due to changes in menstrual cycle | Discontinued previous method due to changes on menstrual cycle (vs. did not)  *SD*  *Main effect* | **0.056**  *0.262*  *-0.026* | **-0.030**  *-0.063*  *-0.072* | **0.143**  *0.586*  *0.020* | **0.202** |  |  |
| **Interactions with frequency of use (on-demand option)**  [the following interaction terms were non-significant in the CLM model and thus were not entered into MML models: Age group; Being in a relationship (vs. not)] | | | | | | |  |
| Vaginal ring use experience | Ever used a ring (vs. not)  *SD*  *Main effect* | **0.057**  *-0.175*  *0.014* | **-0.028**  *-0.402*  *-0.023* | **0.141**  *0.053*  *0.051* | **0.190** |  |  |
| Multiple sexual partners | Two or more partners in last 12 months (vs. 1 or none)  *SD*  *Main effect* | **0.249**  *0.003*  *0.233* | **0.035**  *-0.039*  *0.028* | **0.462**  *0.045*  *0.438* | **0.022** |  |  |

**Supplemental Table 4. Unadjusted and adjusted associations with greater likelihood of using the nonhormonal MPT ring product (n=2,105)**

| *(reference category for variables below is “not”/“does not”/”did not” unless otherwise specified)* | **OR (95% CI)** | **aOR controlling for all other variables in final model^a^ (95% CI)** |
| --- | --- | --- |
| **Younger age** (18-29 vs. 30-49 years) | 1.58 (1.35, 1.85) *** | -- |
| **Has children** | 1.89 (1.61, 2.22) *** | 1.69 (1.43, 2.00) *** |
| **Relationship status**  Not in a relationship (ref)  Unmarried, in a relationship  Married | Overall: 1.20 (1.08, 1.34) ***  --  1.12 (0.92, 1.34)  1.44 (1.16, 1.79) *** | -- |
| **Black/African American** | 1.24 (1.04, 1.46) * | -- |
| **Education completed**  Some high school or less (ref)  High school diploma or GED  Associates or Bachelors degree  Masters, Doctoral, or other Professional degree | Overall: 1.21 (1.08, 1.36) **  --  2.21 (1.49, 3.28) ***  2.28 (1.53, 3.40) ***  3.03 (1.88, 4.87) *** | -- |
| **Rurality**  Rural (ref)  Suburban  Urban | Overall: 1.21 (1.09, 1.34) **  --  1.06 (0.87, 1.29)  1.44 (1.16, 1.78) ** | 1.15 (1.03, 1.29) *  --  1.06 (0.86, 1.30)  1.32 (1.05, 1.64) * |
| **Strong preference for hormonal contraception** | 1.71 (1.45, 2.02) *** | 1.71 (1.44, 2.04) *** |
| **HIV worry** | 1.82 (1.51, 2.18) *** | 1.39 (1.09, 1.77) ** |
| **STI worry** | 1.71 (1.45, 2.02) *** | 1.28 (1.03, 1.60) * |
| **Seeking care for an STI** in the last year | 1.57 (1.27, 1.92) *** | 1.26 (1.02, 1.56) * |
| **Diagnosed with an STI** in the last year | 1.38 (1.05, 1.82) * | -- |
| **Bacterial vaginosis** (several/many times) | 1.39 (1.07, 1.80) * | -- |
| **History of heavy menses** | 1.24 (1.06, 1.45) ** | 1.21 (1.02, 1.43) ** |
| **Discontinued contraceptive method due to side effects** | 1.38 (1.17, 1.61) *** | -- |
| **Experience using a vaginal ring** | 2.50 (2.02, 3.09) *** | 1.96 (1.55, 2.48) *** |
| **Knowing others who have used a vaginal ring** | 1.69 (1.42, 2.00) *** | 1.36 (1.13, 1.63) ** |

*p<0.05 **p<0.01 ***p<0.001

OR: Odds Ratio from ordinal logistic regression model, with outcome being greater likelihood of using the nonhormonal MPT-ring as described (with three successive levels: (0) Very unlikely/unlikely/don’t know; (1) Likely; (2) Very likely). aOR: adjusted Odds Ratio from model controlling for all other variables. CI: Confidence Interval.

**^a^**The final adjusted/multivariate model was determined based on backward elimination, sequentially removing each non-statistically significant variable with the highest p-value, until all variables in the model were statistically significant.

Additional variables assessed in bivariate analyses but with nonsignificant associations include: race categories other than Black/African American; ethnicity (Hispanic/non-Hispanic); income; US region; history of unintended pregnancy; discontinuing a contraceptive method due to unwanted effects on menses; having multiple male sexual partners in the last year.
